# Focal Cerebral Arteriopathy in a Young Adult

**DOI:** 10.7759/cureus.104330

**Published:** 2026-02-26

**Authors:** Prince Pekyi-Boateng, Lee S Chung, McKenna L Coletti, Lisa M Pabst, Ramesh Grandhi

**Affiliations:** 1 Neurology, University of Utah, Salt Lake City, USA; 2 Pediatrics, University of Utah, Salt Lake City, USA; 3 Neurosurgery, University of Utah, Salt Lake City, USA

**Keywords:** acute ischemic stroke (ais), cerebral angiogram, focal cerebral arteriopathy (fca), stroke mimics, supraclinoid artery stenosis

## Abstract

Focal cerebral arteriopathy (FCA) is a rare, monophasic stenosis of the distal internal carotid artery (ICA) or proximal middle cerebral artery that primarily affects children but is occasionally seen in adults. It is unclear what causes FCA in adults, which complicates diagnosis and management. The case presented highlights diagnostic challenges using multimodal imaging, supporting conservative management, and the need for adult-specific treatment criteria. A 38-year-old woman with a history of breast cancer, migraines, and prior pulmonary embolism presented with a sudden-onset thunderclap headache and expressive aphasia. Initial imaging revealed high-grade left ICA terminus stenosis, with differential diagnoses including reversible cerebral vasoconstriction syndrome and vasculitis. The patient was readmitted two days after her initial presentation for worsening symptoms, including right-sided facial and arm numbness, as well as visual hallucinations. Multimodal imaging, blood work, and lumbar puncture excluded alternative diagnoses. Digital subtraction angiography confirmed 70% left supraclinoid ICA stenosis that was unresponsive to intra-arterial verapamil, ruling out reversible cerebral vasoconstriction syndrome. Serial imaging demonstrated progressive improvement in the stenosis (59% at one month and 46% at six months). The patient has not experienced symptom recurrence and reports being at her neurologic baseline. This case underscores the challenges of diagnosing FCA in young adults because of its overlap with several other arteriopathies, resulting in unnecessary interventions and lifelong misdiagnosis. Multimodal imaging is critical for accurate diagnosis and monitoring. The self-limiting nature of FCA supports conservative management, but adult-specific diagnostic criteria are needed to improve recognition and guide therapy.

## Introduction

Focal cerebral arteriopathy (FCA) is a rare cause of cerebrovascular insufficiency in young adults characterized by monophasic stenosis of the distal internal carotid artery (ICA) or proximal middle cerebral artery (MCA). The monophasic nature of FCA means the arterial abnormality is acute and self-limited. It may exhibit brief worsening during the initial days to weeks but thereafter follows a nonprogressive course. On follow-up imaging, the stenosis typically stabilizes, partially improves, or resolves completely, without evidence of persistent, recurrent, or progressive inflammatory changes after the initial phase. With an incidence of ~2.8 cases per 1,000 person-years [1], it is less common in adults than in children, where it accounts for ~25% of strokes that are assessed with vascular imaging [2]. Its overlapping clinical and imaging features often lead to misdiagnosis as atherosclerosis, primary central nervous system vasculitis, arterial dissection, or reversible cerebral vasoconstriction syndrome (RCVS), potentially leading to overtreatment [3,4]. FCA has been linked to viral infections like varicella-zoster virus, but this link may go unrecognized without regular virological testing [2]. Pediatric FCA is well characterized with established diagnostic criteria [5,6], but the diagnosis of adult cases remains challenging. However, high-resolution magnetic resonance imaging (HR-MRI) and magnetic resonance angiography (MRA) aid diagnosis, and digital subtraction angiography (DSA) is the gold standard because of its superior resolution [3,4].

FCA typically presents in adults as a unifocal, unilateral disease with often nonprogressive stenosis. In inflammatory FCA, high-resolution vessel wall imaging typically reveals concentric vessel wall thickening with avid contrast enhancement, indicating an active inflammation process. In contrast, noninflammatory FCA (such as dissection-related or other noninflammatory subtypes) shows no significant enhancement or notable wall thickening on imaging [7,8]. Unlike inflammatory cases, which may benefit from antivirals when a viral cause is likely [6], noninflammatory FCA does not respond to steroids. Per the American Heart Association/American Stroke Association guidelines, antiplatelet therapy (e.g., aspirin) is recommended for noninflammatory FCA to prevent stroke or transient ischemic attack (TIA) recurrence, and anticoagulation is recommended for high-risk thromboembolic cases [9]. Adults with FCA often experience recurrent TIA or minor strokes in a single vascular territory.

Three case series underscore the role of FCA in arterial ischemic stroke in adults. Bulder et al. [3] studied 30 young adults in the Netherlands, noting frequent patient improvement, some with complete normalization, and few recurrences. Similarly, McKenna et al. [4] examined 16 Irish young adults, finding that FCA often manifests as recurrent TIAs or minor strokes, with most patients achieving favorable outcomes and arteriopathy stabilization or resolution. Third, Bhasi et al. [1] reported a seven-year retrospective case series from an Indian tertiary care center, identifying six adult patients with FCA. HR-MRI confirmed diagnoses by detecting eccentric arterial wall thickening and enhancement that distinguished FCA from vasculitis. Serial HR-MRI showed arteriopathy stabilization without progression or recurrence.

This case report and literature review provide a detailed analysis of FCA in a young adult, using multimodal serial imaging to confirm diagnosis after an initial misdiagnosis, and document the ultimate necessity of serial DSA in diagnosis. Unlike the broader studies that focus on group outcomes and arteriopathy stabilization, this report emphasizes a single adult case with robust evidence to exclude mimics, address misdiagnosis, and prevent overtreatment and underscores the need for adult-specific diagnostic criteria to enhance FCA recognition and management in young adults.

## Case presentation

**Figure 1:**
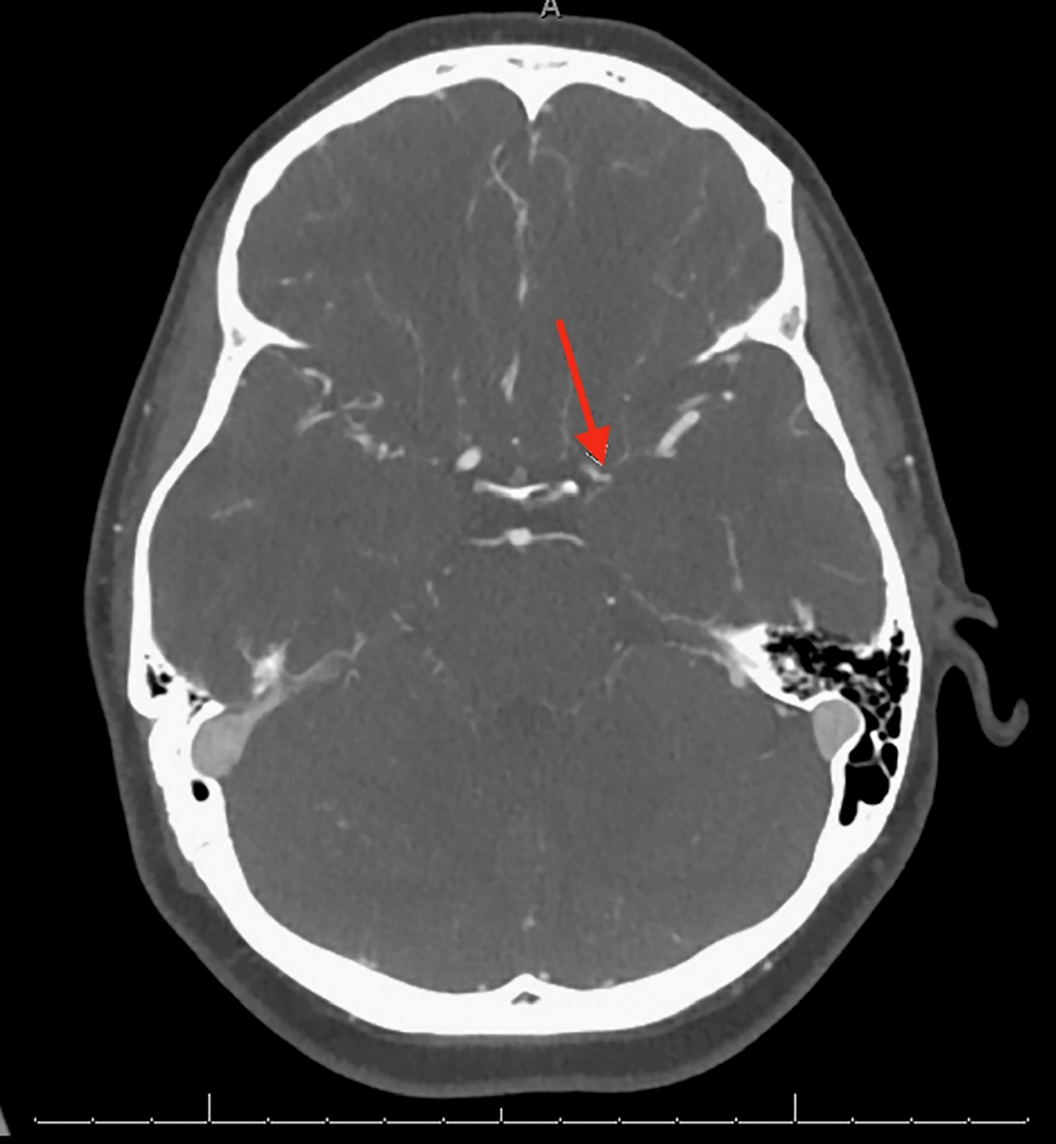
Computed tomography angiography of the head in a patient presenting with sudden-onset thunderclap headache and expressive aphasia The arrow indicates persistent focal high-grade stenosis of the distal left supraclinoid internal carotid artery (ICA) of at least 50% according to the Warfarin–Aspirin Symptomatic Intracranial Disease (WASID) study criteria.

A 38-year-old woman with a history of breast cancer who had undergone bilateral mastectomy and was taking anastrozole after chemotherapy presented with a sudden-onset thunderclap headache and word-finding difficulties. Her medical history also included migraines, psoriasis, and a prior pulmonary embolism managed with apixaban. Computed tomography angiography (CTA) revealed intracranial arterial stenosis that was most pronounced at the left ICA terminus (Figure 1). The differential diagnoses included RCVS and vasculitis. She was admitted for further evaluation and discharged with close follow-up after symptom management. Two days later, she returned with worsening headaches, new right-sided facial and arm numbness, and visual hallucinations.

**Figure 2:**
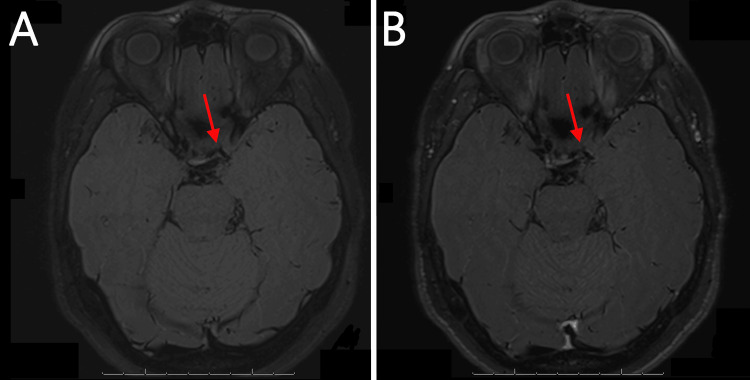
Magnetic resonance imaging (MRI) with vessel wall imaging (A) Precontrast and (B) postcontrast delays alternating with nutation for tailored excitation (DANTE) MRI sequences demonstrated ≥60% stenosis (WASID study criteria) of the left distal ICA (arrows) with associated hyperintensity of the vessel wall but no gadolinium enhancement. WASID: Warfarin–Aspirin Symptomatic Intracranial Disease; ICA: Internal carotid artery.

Given her RCVS score of 4 (intermediate risk) and her history of chemotherapy and sumatriptan use, RCVS was initially considered the likely cause of her symptoms. She was started on 120 mg of verapamil daily for RCVS-related headaches, but this was discontinued when she did not tolerate the first few doses. A lumbar puncture revealed normal opening pressure, a negative infection panel, no pleocytosis, no elevation in protein levels, and unremarkable flow cytometry or cytology, making vasculitis and infection unlikely. MRI with vessel wall imaging demonstrated ≥60% stenosis of the left distal ICA with associated hyperintensity of the vessel wall on both pre- and postcontrast delay alternating with nutation for tailored excitation (DANTE) MRI sequence but no gadolinium enhancement (Figure 2, Panels A and B). The absence of enhancement and high-risk features made both a vulnerable atherosclerotic plaque and inflammation due to primary angiitis of the central nervous system unlikely. Serial transcranial Doppler (TCD) studies over a week showed persistent elevation of left MCA velocities, with a significantly elevated Lindegaard ratio suggesting moderate-to-severe vasospasm.

**Figure 3:**
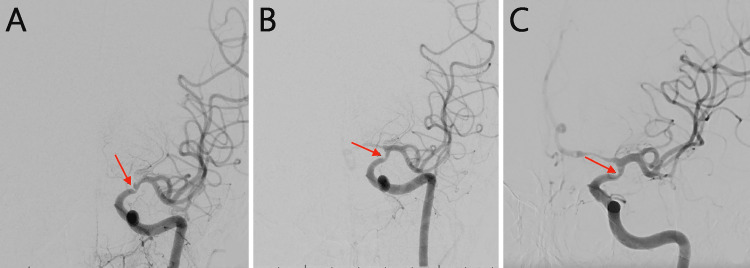
Cerebral angiograms (A) The arrow indicates focal stenosis of the left ICA terminus before the ICA bifurcation measuring 70% stenosis (WASID study criteria) (measuring 0.7 mm, proximal segment 2.4 mm, and distal segment 2.5 mm), with no change in vessel caliber after 10 mg of verapamil was injected into the patient’s left ICA. (B) The arrow indicates improved left ICA terminus stenosis, now measuring 59% and demonstrating improved flow through the left ICA, with flash filling of the ipsilateral A1 and A2 ACA segments that were not seen on prior angiograms. (C) The arrow indicates improvement in the known clinoid segment stenosis just proximal to the ICA bifurcation at the left ICA terminus, measuring 46% (compared with the previous 59%). There is washout filling of the ipsilateral A1 and distal ACA segments. ICA: Internal carotid artery; WASID: Warfarin–Aspirin Symptomatic Intracranial Disease; ACA: Anterior cerebral artery.

DSA demonstrated significant focal stenosis (70%) of the left ICA terminus, just proximal to the ICA bifurcation (Figure 3, Panel A). Collateral circulation was noted, with good filling of the left anterior cerebral artery (ACA) and MCA branches from the contralateral ICA via the anterior communicating artery. The patient was given 10 mg of verapamil, injected intra-arterially in the left ICA, resulting in no change in vessel caliber on post-verapamil angiograms, making the diagnosis of RCVS less likely.

With symptom resolution and improvement in headaches, the patient was discharged with close monitoring. No anti-thrombotic medication or lipid-lowering medications were newly prescribed upon discharge. By adapting the pediatric Focal Cerebral Arteriopathy Scoring System tool (descriptively rather than diagnostically), we could categorize the patient’s condition as mild FCA requiring conservative management because most cases in this category are monophasic, self-limiting, and have a favorable natural history [10]. A repeat diagnostic cerebral angiogram performed two months after discharge demonstrated improvement in the patient’s left ICA terminus stenosis from 70% to 59%. The re-imaging showed improved flow through the left ICA, with flash filling of the ipsilateral A1 and A2 ACA segments, which had not been visualized on prior studies (Figure 3, Panel B). A six-month follow-up cerebral angiogram revealed further improvement (to 46%) of the known clinoid segment stenosis just proximal to the left ICA bifurcation (Figure 3, Panel C). During follow-up, the patient reported full resolution of prior neurological deficits and headaches, and DSA demonstrated progressive improvement of the focal stenosis, most likely consistent with FCA in an adult.

This patient will undergo continued surveillance with imaging with either CTA or DSA every six months until she is stable or there is full resolution of the stenosis.

## Discussion

FCA in adults is challenging to diagnose because of its monophasic stenosis, typically affecting the distal ICA or proximal MCA. FCA mimics conditions like atherosclerosis, central nervous system vasculitis, fibromuscular dysplasia, arterial dissection, and RCVS [3,4,11,12]. Symptoms such as recurrent TIAs, minor strokes in one vascular territory, or sudden severe headaches are suggestive but nonspecific [3,4].

Risk factors are less clear in adults than in children, where FCA is often postinfectious and unilateral [2,5,6,8]. In adults, potential triggers include viral infections (e.g., varicella-zoster virus), autoimmune disorders, or prothrombotic states, but many cases are idiopathic [3,4]. Adult FCA may involve genetic or acquired vasculopathies, with weaker links to infections [3,4]. Treatment is complicated by limited evidence. The American Heart Association/American Stroke Association recommends antiplatelet therapy (e.g., aspirin) for noninflammatory FCA [9], whereas inflammatory cases may require treatment with antivirals or corticosteroids [6]. The self-limiting nature of FCA often favors conservative management over interventions like stenting, but the risk of inappropriate treatment remains because of the difficulty in distinguishing subtypes, especially in adults with comorbidities [3,4,9].

Imaging is critical for differentiating FCA from other arteriopathies. TCD is useful for monitoring disease progression but is less specific and is operator-dependent compared with HR-MRI or DSA [10]. In patients with hypertension or hyperlipidemia, atherosclerosis typically shows eccentric wall thickening and calcification on imaging, and HR-MRI often reveals eccentric vessel wall enhancement and intraplaque hemorrhage [12]. Patients with vasculitis, whether autoimmune or infectious, may present with symptoms, including cognitive impairment and headaches, and HR-MRI will demonstrate segmental, concentric, homogeneous vessel wall enhancement involving multiple vascular segments with diffuse narrowing [12]. Lumbar puncture is essential to rule out autoimmune diseases or infections [11,12]. Imaging in patients with angiitis, similar to that of patients with vasculitis, displays a “beading” pattern of alternating stenosis and dilation that is absent in FCA [13]. Fibromuscular dysplasia is more common in female patients, who present with headaches, pulsatile tinnitus, and the “string of beads” appearance on imaging [14]. Patients with arterial dissection, which is often trauma-related, show intimal flaps and intramural hematomas on HR-MRI that are absent in FCA [15].

Patients with RCVS that is triggered by stress or medications have negligible-to-mild vessel wall enhancement and uniform thickening on HR-MRI, often with convexity subarachnoid hemorrhage [11,16]. The RCVS2 clinical score (≥5) has a high sensitivity (90%) and specificity (99%) for diagnosis [14]. Intra-arterial calcium channel blockers like verapamil can reverse RCVS but are less effective for FCA. A ≥32% change in arterial caliber after intra-arterial verapamil has a high sensitivity (100%) and specificity (88.2%) for RCVS [17].

In this patient’s case, DSA revealed 70% stenosis in the left supraclinoid ICA terminus, with collateral flow to the left ACA and MCA and minor right M1 MCA irregularity [3]. Serial DSA showed stenosis improvement to 59% at one month and 46% at six months, consistent with FCA’s self-resolving course [4]. ICA stenosis can alter the circle of Willis distal vessel wall irregularities because of hemodynamic shifts, potentially leading to ischemia if the vascular reserve is depleted, as indicated by elevated TCD velocities [13,18].

Intracranial stenting is not recommended as therapy for FCA. Stenting may be considered only in highly selected patients with severe (70%-99%) stenosis who have recurrent stroke or TIA despite optimal medical therapy, based primarily on whether the patient has intracranial atherosclerotic disease (ICAD) rather than FCA [19,20]. Angioplasty, however, may be beneficial among a specific group of symptomatic patients, but there is not enough data to support its safety and benefits in the current literature.

Diagnosis of FCA requires a multimodal approach: CTA or MRA for stenosis detection, HR-MRI for vessel wall analysis [7,14], lumbar puncture to exclude vasculitis or infection [11], and DSA for precise assessment [3,4,8]. Mild cases warrant conservative management with antiplatelet therapy [9]; antivirals or corticosteroids can be reserved for inflammatory cases [6], with follow-up imaging to confirm resolution [4].Pediatric tools like the Focal Cerebral Arteriopathy Scoring System may aid adult prognostication [10], but adult-specific guidelines are needed [3,4].

This report employs multimodal imaging (CTA, HR-MRI, DSA, and TCD) to showcase stenosis improvement through comprehensive serial imaging, integrated with prior studies, in the distinctive case of a 38-year-old patient with unique symptoms. Our approach blends diverse imaging techniques and detailed longitudinal data to provide valuable insights into a rare clinical presentation, enhances real-time follow-up and assessment, and aids in ruling out diagnostic mimics. However, the single-case design, the patient’s distinct profile, and a limited six-month follow-up period pose notable limitations.

## Conclusions

FCA in young adults is a rare, underrecognized cause of ischemic stroke that is often mistaken for other cerebrovascular diseases. Diagnosis requires multimodal imaging to detect monophasic stenosis and distinguish between inflammatory and noninflammatory subtypes. Tailored management and follow-up imaging are crucial, given FCA’s typically self-limiting course and favorable prognosis.
